# School and work absenteeism due to menstruation in three West African countries: findings from PMA2020 surveys

**DOI:** 10.1080/26410397.2021.1915940

**Published:** 2021-05-09

**Authors:** Julie Hennegan, Funmilola M. OlaOlorun, Sani Oumarou, Souleymane Alzouma, Georges Guiella, Elizabeth Omoluabi, Kellogg J. Schwab

**Affiliations:** a Research Associate, The Water Institute, Department of Environmental Health and Engineering, Johns Hopkins Bloomberg School of Public Health, Baltimore, MD, USA. *Correspondence*: julie.hennegan@burnet.edu.au; b Senior Lecturer, Department of Community Medicine, College of Medicine, University of Ibadan, University College Hospital, Ibadan, Nigeria; c Statisticien Démographe, Conseiller du Directeur Général, l’Institut National de la Statistique du Niger, Niamey, Niger; d Ingénieur Statisticien Economiste, Directeur des Enquêtes et des Recensements, l’Institut National de la Statistique du Niger, Niamey, Niger; e Lecturer, Institut Supérieur des Sciences de la Population, University of Ouagadougou, Ouagadougou, Burkina Faso; f Senior Lecturer, Department of Statistics, University of the Western Cape, Cape Town, South Africa; g Professor, The Water Institute, Department of Environmental Health and Engineering, Johns Hopkins Bloomberg School of Public Health, Baltimore, MD, USA

**Keywords:** menstrual hygiene, menstrual health, dysmenorrhea, survey, school attendance, employment

## Abstract

Reports of school and work absences due to unmet menstrual needs have prompted increased attention to menstruation in policy and practice. However, there appear to be few quantitative studies reported in published literature capturing the prevalence of this hypothesised absenteeism. This study undertook secondary analysis of nationally representative Performance Monitoring and Accountability 2020 (PMA2020) data from Burkina Faso and Nigeria, and city-representative data from Niamey, Niger to determine the extent of women’s and girls’ self-reported absence from school and work due to menstruation. Among women and adolescent girls aged 15–49 years who had worked outside the household in the past month in Burkina Faso (*n *= 998), Niger (*n *= 212) and Nigeria (*n *= 3638), 19%, 11% and 17%, respectively, reported missing work due to menstruation. Among those aged 15–24 years who attended school in the past year in Burkina Faso (*n *= 461), Niger (*n *= 213) and Nigeria (*n *= 1574), 17%, 15% and 23% reported missing school in the past year due to menstruation. Findings support the assertion that menstruation is a source of absenteeism in West Africa and indicate that greater attention from research, practice, and policy is needed. In presenting this data we also reflect critically on the performance of questions regarding menstrual-related absenteeism in national monitoring surveys. Future monitoring efforts should consider the interpretability of similar survey data when many respondents did not attend any school or work and were ineligible to answer questions regarding absenteeism. Further, without additional research identifying the reasons for absenteeism, findings from similar survey questions may be difficult to interpret with relevance for policy decision making.

## Résumé

Des informations faisant état d’absences à l’école et au travail en raison de besoins menstruels non satisfaits ont incité à accorder davantage d’attention aux menstruations dans les politiques et la pratique. Néanmoins, il semble y avoir peu d’études quantitatives mentionnées dans les publications qui évaluent la prévalence de cet absentéisme hypothétique. Cette étude a entrepris une analyse secondaire de données de la PMA2020 (Performance Monitoring and Accountability 2020) représentatives au niveau national pour le Burkina Faso et le Nigéria, et représentatives d’une zone urbaine (Niamey) pour le Niger, afin de mesurer les absences à l’école et au travail du fait des menstruations notifiées par les femmes et les jeunes filles elles-mêmes. Parmi les femmes et les adolescentes âgées de 15 à 49 ans qui avaient travaillé en dehors du foyer pendant le mois passé au Burkina Faso (*n *= 998), au Niger (*n *= 212) et au Nigéria (*n *= 3638), 19, 11 et 17% respectivement ont indiqué avoir manqué le travail en raison de leurs menstruations. Parmi les femmes âgées de 15 à 24 ans qui avaient fréquenté l’école au cours de l’année précédente au Burkina Faso (*n *= 461), au Niger (*n *= 213) et au Nigéria (*n *= 1574), 17, 15 et 23% ont rapporté avoir manqué les cours pendant l’année écoulée du fait de leurs menstruations. Les résultats étayent l’hypothèse selon laquelle la menstruation est une source d’absentéisme en Afrique de l’Ouest et indiquent que la recherche, la pratique et les politiques doivent accorder plus grande attention à ce problème. Parallèlement à la présentation de ces données, nous réfléchissons aussi de manière critique à la performance des questions concernant l’absentéisme lié aux menstruations dans les enquêtes nationales de suivi. Les futures activités de suivi devraient tenir compte de l’interprétabilité de données d’enquêtes similaires quand beaucoup de répondantes ne sont pas allées à l’école ni travailler et ne réunissaient donc pas les conditions pour répondre aux questions concernant l’absentéisme. En outre, sans recherches complémentaires identifiant les raisons de l’absentéisme, les conclusions d’enquêtes similaires risquent d’être difficiles à interpréter pour guider la prise de décisions politiques.

## Resumen

Informes de ausencias escolares y laborales debido a necesidades menstruales insatisfechas han generado mayor atención a la política y práctica relacionadas con la menstruación. Sin embargo, en la literatura publicada hay pocos estudios cuantitativos que capturen la prevalencia de este ausentismo hipotético. **E**ste estudio realizó un análisis secundario de los datos de Monitoreo y Rendición de Cuentas del Rendimiento 2020 (PMA2020) representantes de las naciones de Burkina Faso y Nigeria, y datos representantes de la ciudad de Niamey, en Níger, para determinar el alcance de las ausencias escolares y laborales autoinformadas por mujeres y niñas debido a la menstruación. Entre las mujeres y adolescentes de 15 a 49 años que habían trabajado fuera del hogar en el último mes en Burkina Faso (*n *= 998), Níger (*n *= 212) y Nigeria (*n *= 3638), el 19, el 11 y el 17% respectivamente, informaron faltar al trabajo debido a la menstruación. Entre aquéllas de 15 a 24 años que asistieron a la escuela en el último año en Burkina Faso (*n *= 461), Níger (*n *= 213) y Nigeria (*n *= 1,574), el 17, el 15 y el 23% informaron haber faltado a la escuela en el último año debido a la menstruación. Los hallazgos corroboran la afirmación de que la menstruación es una causa de ausentismo en África occidental e indican que se necesita mayor atención a este asunto en las investigaciones, la práctica y la política. Al presentar estos datos también reflexionamos críticamente sobre el rendimiento de las preguntas sobre el ausentismo relacionado con la menstruación en las encuestas de monitoreo nacional. Futuros esfuerzos de monitoreo deben considerar la interpretabilidad de datos de encuestas similares cuando muchas encuestadas no asistieron a la escuela o al trabajo y no fueron elegibles para contestar preguntas sobre su ausentismo. Sin realizar más investigaciones para identificar las razones del ausentismo, podría ser difícil interpretar los hallazgos de preguntas de encuestas similares con importancia para la toma de decisiones sobre políticas.

## Introduction

A cornerstone of advocacy for the importance of menstruation in the lives of girls in the Global South has been the claim that unmet menstrual needs prevent them from attending school. This impact on education has prompted the mobilisation of funds and large-scale policies such as free menstrual product provision in multiple countries, including Ghana^1^ and Kenya.^2^ Despite increasing action, we identified few high-quality studies assessing girls’ absences during menstruation or testing the effectiveness of the suggested policy and programme responses.^3^ The oft-cited statistic that one in ten girls in Africa miss school during menstruation has persisted, despite frequent efforts to highlight that this “zombie statistic” is unsubstantiated.^4,5^

A large body of qualitative studies support the hypothesis that girls miss school during menstruation and have identified a range of barriers to school attendance and engagement, including: inadequate clean and reliable menstrual materials, unsupportive water and sanitation infrastructure, lack of disposal facilities, insufficient information about menstruation, poor social support, and pervasive menstrual stigma.^6–10^ Further, menstrual pain and fatigue have often been identified as prominent reasons for missing school.^8,10,11^ We found few quantitative studies in the published literature reporting the level of school absenteeism due to menstruation. One national survey in Bangladesh in 2013 found that 41% of menstruating girls aged 11–17 reported missing school during menstruation,^12^ while a survey across four provinces in Indonesia reported that 11% of girls aged 12–19 years had missed school during their last period.^13^ In Nigeria, a cross-sectional study of 583 female university students found 82% reported dysmenorrhea and 43% reported missing school due to menstrual pain, although the time period was unclear,^14^ while in Ghana, another study of undergraduates found 84% reporting dysmenorrhea, with 61% reporting this influenced daily activities.^15^

The impact of menstruation on the lives of adult women has received far less attention, with acknowledgements that unsupportive environments and stigma may result in absenteeism from formal or informal employment and create challenges for women at home.^3,16–18^ Across contexts, menstruation is frequently constructed as “dirty” or “impure”, with women and adolescent girls expected to hide evidence of menstruation or to restrict activities such as cooking during menses.^10,19,20^

More recently, Multiple Indicator Cluster Surveys (MICS) have captured women’s and girls’ (aged 15–49 years) reports of absence from school, work, or social activities due to menstruation in the past year. MICS data reports a combined proportion, representing non-participation in any of these activities due to menstruation. In data collections undertaken to date, 20% of respondents in Sierra Leone in 2017,^21^ 19% in Ghana in 2018,^22^ 16% in Zimbabwe in 2019,^23^ 20% in The Gambia in 2019, and 8% in Bangladesh in 2019^24^ reported non-participation due to menstruation.

These new monitoring efforts respond to calls to capture the state of unmet menstrual health needs across populations and to monitor progress as policy and programming are implemented. However, to date there are no established indicators for monitoring menstrual health and the inadequacy of measures for core menstrual health constructs has frequently been highlighted as a priority research gap.^25–28^ The construction of indicators and questions used in recent national monitoring efforts including MICS and the Performance Monitoring and Accountability (PMA2020) survey data used in this study present opportunities to interrogate the performance of measures being implemented to identify strengths and limitations and recommend improvements.

The objectives of this study were: (1) to describe the extent of women’s and girls’ self-reported school and work absenteeism due to menstruation in three countries: Burkina Faso, Nigeria, and Niamey, Niger; (2) to describe absenteeism across key socio-demographic groups; and (3) through discussion, to reflect on the performance of the questions used in the PMA2020 surveys and highlight considerations for the interpretation of the results and use of similar questions in future surveys.

## Methods

We undertook secondary analysis of Performance Monitoring and Accountability (PMA2020) survey data.^29^

### PMA2020

The PMA2020 survey programme was initiated following the 2012 London Summit on Family Planning and relies on cohorts of resident enumerators and smartphone survey data collection to monitor country progress towards achieving family planning goals. The nationally, or regionally, representative surveys are undertaken in 11 countries once or twice per year, with data made publicly available rapidly following survey completion. The surveys include a focus on family planning, as well as household water, sanitation and hygiene. Since 2015, PMA2020 has collected national data on menstrual health and hygiene in selected data collection rounds in each country (approximately one in every five or six surveys). In 2018, new questions related to absence from school and work during menstruation were added and are presented in this study.

For all surveys, national statistical agency master sampling frames were used to draw enumeration areas (EA). Households were mapped and listed in each EA and then randomly selected for participation using a “Random Number Generator” application. Within each household, all females aged 15–49 years were contacted and invited to participate in the female questionnaire. Interviews were conducted by enumerators using a smartphone app. Enumerators were females fluent in local languages, who resided in or near the selected enumeration areas, and were trained to conduct PMA2020 surveys. Interviews were conducted with auditory and visual privacy. Enumerator training included the sensitive nature of menstrual questions and the question set was preceded by a prompt to reiterate that responses were anonymous and confidential. Only respondents who had menstruated within the past three months were asked questions about their menstrual practices and absenteeism. Data collection dates and sampling information for each country are provided below. We report the total number of households and females included in the survey. The analytic sample of respondents included in the present study (that is, female household residents eligible to answer questions about menstruation and who provided valid responses to school or work questions), is reported in results. The characteristics of this sub-sample compared to the full population of women across PMA2020 surveys are discussed in more detail elsewhere.^30^ Further details regarding the PMA2020 surveys are published elsewhere,^29^ and available on the survey programme website.

### Surveys

#### Burkina Faso^31^

Data from the fifth round of PMA2020 surveys in Burkina Faso, collected between November 2017 and January 2018, were used for this study. This survey used a two-stage sampling approach in which 83 EAs were drawn from the Institut National de la Statistique et de la Démographie master sampling frame from 2006. In each EA, 35 households were randomly selected. The final sample included a total of 2811 households and 3556 females.

#### Niger^32^

Data from the fifth round of PMA2020 surveys in Niamey, Niger, collected between June and August 2018 in Niamey, were used for this study. A total of 33 EAs were drawn using probability proportional to size from a sampling frame provided by the Fourth General Census of Population and Housing conducted by Niger’s National Statistics Institute from 2012. In each EA, 35 households were randomly selected. The final sample included 1089 households, and 1281 females.

#### Nigeria^33^

Data from the fifth round of PMA2020 surveys in Nigeria, collected between April and May 2018, were used for this study. This survey used a three-stage sampling approach using a sample of seven states. One state per zone was selected using probability proportional to size from among each of Nigeria’s six zones. The seventh state (Kaduna) was allocated to the northwest zone and zonal weights were adjusted accordingly. A total of 302 clusters of enumeration areas (EAs) were drawn from the National Population Commission’s Census master sampling frame from 2006. In each cluster of EAs, 35 households (40 in Lagos) were randomly selected. The final completed sample included 10,070 households and 11,106 females. Weights were applied to make the data nationally representative.

### Measures

Household and female questionnaires are publicly available on the PMA2020 website (www.pma2020.org/questionnaires).

#### Work absenteeism

All eligible respondents aged between 15 and 49 years were asked “Aside from your own housework, have you done any work in the last month?” with “Yes” or “No” response options. For those who reported having undertaken work in the past month, they were asked “Due to your last menstrual period, were there any work days in the last month that you did not attend?” with Yes/No responses.

#### School absenteeism

Eligible survey respondents between 15 and 24 years of age were asked “Did you attend school any time in the past 12 months” with Yes/No response options. Those who had attended school were then asked: “Due to your menstrual period, were there any school days in the past 12 months that you did not attend?”

#### Demographic characteristics

Wealth tertiles were calculated for each survey population according to data reported for the household on items such as building materials of the home, asset ownership and water and sanitation facilities in the household. Urban and rural residence were determined by country statistical agencies according to the household location. Survey participants reported their age in years, marital status and highest level of schooling attended.

#### Menstrual materials

To capture the materials used as menstrual absorbents, participants were asked: “During your last menstrual period, what did you use to collect or absorb your menstrual blood?” This was a multi-response question and enumerators prompted respondents for all the materials used, asking if there was “anything else?” For the present study, results are presented for those who reported using sanitary pads, cloth, and the next most prevalent product in the country. This product was: underwear alone in Burkina Faso, cotton wool in Niger, and toilet paper in Nigeria. In addition, an “other” category was included, grouping the remaining menstrual absorbents in each country (e.g. cotton wool, a bucket, toilet paper). We used binary variables for each material (used vs not used) for descriptive and multivariable analyses.

### Analyses

Quantitative analyses were undertaken using Stata 14. All analyses were adjusted for the complex survey design using the female sampling weights provided in PMA2020 data sets. Descriptive statistics report the proportion of surveyed women and girls who reported undertaking work outside the home and attending school in the respective time periods according to demographic characteristics and the type of menstrual material used. Of those who attended any school or work, the proportion absent due to their menstrual period is reported. Binary and multiple logistic regressions were used to compare self-reported absence from school or work according to demographic predictors. All sociodemographic data were retained in multiple regressions, along with the main two menstrual materials used (pads and cloth). Due to the small sample of respondents who were asked relevant questions in Niger, smaller demographic groupings were used for some analyses for these data.

## Results

### Respondents

The present study included survey respondents eligible to answer questions about menstruation. That is, female household residents who reported menstruating in the past three months, and who were 15–49 years of age.^30^ This resulted in an analytic sample of 2468 respondents in Burkina Faso (69% of the female questionnaire sample), 978 respondents in Niamey, Niger (76% of the female questionnaire sample), and 8360 respondents in Nigeria (78% of the female questionnaire sample). Sample characteristics for each geography are reported in Table 1.

**Table 1.** Study sample characteristics

	Burkina Faso (*n *= 2468) *n* (%)	Niamey, Niger (*n *= 978) *n* (%)	Nigeria (*n *= 8360) *n* (%)
**Age**			
15–19	636 (25.78)	283 (28.91)	1832 (21.91)
20–24	392 (15.88)	167 (17.12)	1310 (15.67)
25–29	57 (14.44)	151 (15.42)	1467 (17.55)
30–34	342 (13.87)	144 (14.73)	1185 (14.17)
35–39	304 (12.31)	107 (10.97)	1164 (13.92)
40–49	437 (17.72)	126 (12.85)	1402 (16.77)
**Marital status**			
Married/Cohabitating	1690 (68.46)	523 (53.47)	4669 (55.86)
Divorced or widowed	99 (4.03)	75 (7.72)	434 (5.19)
Never married	679 (27.51)	380 (38.82)	3256 (38.95)
**Education**			
Never	1429 (57.94)	247 (25.27)	1167 (13.96)
Primary	416 (16.88)	196 (20.10)	1120 (13.40)
Secondary	568 (23.03)	417 (42.69)	4212 (50.38)
Higher	53 (2.16)	117 (11.95)	1861 (22.26)
**Wealth tertile**			
Lowest tertile	820 (33.21)	274 (28.04)	2126 (25.43)
Middle tertile	778 (31.51)	324 (33.09)	2834 (33.91)
Highest tertile	871 (35.27)	380 (38.87)	3400 (40.66)
**Residence**			
Rural	1853 (75.07)	46 (4.72)	3235 (38.70)
Urban	615 (24.93)	932 (94.28)	5124 (61.30)

### Work and school participation

The proportion of respondents who attended school and work, and those who subsequently reported absences due to menstruation, are reported in Table 2 for Burkina Faso, Table 3 for Niger, and Table 4 for Nigeria.

**Table 2.** Self-reported work and school attendance and absenteeism due to menstruation in **Burkina Faso** according to socio-demographic characteristics and menstrual materials used

	Work (past month)				School (past year)			
	Worked (*n *= 2468)	Missed (*n *= 998)	OR (95%CI)*	aOR (95%CI)*	School (*n *= 762)	Missed (*n *= 461)	OR (95%CI)**	aOR (95%CI)**
**Age**								
15–19	23.93	22.73	1.00	1.00	61.41	16.32	1.00	1.00
20–24	28.48	22.82	1.01 (0.49–2.06)	1.33 (0.59–2.96)	45.02	20.18	1.30 (0.71–2.36)	0.98 (0.46–2.11)
25–29	40.53	19.89	0.84 (0.47–1.52)	1.14 (0.50–2.59)				
30–34	48.12	18.42	0.77 (0.39–1.52)	1.02 (0.39–2.64)				
35–39	44.28	13.85	0.55 (0.27–1.10)	0.66 (0.27–1.62)				
40–49	49.20	18.15	0.75 (0.47–1.22)	0.88 (0.44–1.75)				
**Marital status**								
Married/cohabitating	39.80	19.01	1.00	1.00	15.26	19.97	1.00	1.00
Divorced or widowed	58.16	14.60	0.73 (0.38–1.39)	0.94 (0.47–1.90)	22.23	0	–	–
Never married	28.35	21.06	1.14 (0.71–1.81)	1.32 (0.66–2.65)	68.27	17.21	0.83 (0.31–2.22)	0.85 (0.29–2.51)
**Education**								
Never	37.51	20.07	1.00	1.00			–	
Primary	47.80	21.43	1.09 (0.60–1.95)	1.18 (0.65–2.13)	17.66	17.33	1.00	1.00
Secondary	28.11	14.55	0.68 (0.40–1.14)	0.73 (0.40–1.33)	71.92	16.17	0.92 (0.30–2.81)	0.76 (0.24–2.44)
Higher	52.25	12.14	0.55 (0.20–1.48)	0.78 (0.30–2.08)	97.94	36.39	2.73 (0.83–9.01)	1.86 (0.50–6.93)
**Wealth tertile**								
Lowest tertile	33.09	24.04	1.00	1.00	58.33	12.02	1.00	1.00
Middle tertile	33.69	21.60	0.87 (0.51–1.49)	0.85 (0.48–1.48)	42.20	16.64	1.46 (0.60–3.58)	1.67 (0.66–4.20)
Highest tertile	44.83	14.17	0.52 (0.34–0.80)	0.73 (0.45–1.64)	62.65	19.69	1.79 (0.78–4.12)	1.86 (0.68–5.05)
**Residence**								
Rural	33.61	22.01	1.00	1.00	52.33	14.49	1.00	1.00
Urban	48.78	13.28	0.54 (0.32–0.91)	0.75 (0.40–1.39)	63.00	21.60	1.63 (0.91–2.90)	1.36 (0.68–2.74)
Menstrual materials***								
Pads	44.07	14.35	0.57 (0.33–0.97)	0.64 (0.33–1.25)	61.21	17.50	1.01 (0.55–1.85)	0.52 (0.23–1.15)
Cloth	35.37	21.62	1.38 (0.85–2.22)	0.87 (0.46–1.65)	42.27	15.36	0.83 (0.37–1.85)	0.64 (0.27–1.55)
Underwear alone	29.42	21.33	1.16 (0.60–2.24)	^†^	56.44	18.97	1.11 (0.32–3.86)	^†^
Other (e.g. cotton wool, toilet paper)	49.46	17.37	0.88 (0.36–2.14)	^†^	55.35	19.05	1.12 (0.49–2.59)	^†^
**Total**	**37.39**	**19.17**			**56.11**	**17.32**		

*Of respondents who reported working in the past month.

**Of respondents aged 15–24 who reported attending school in the past 12 months.

***Multiple-response variable.

^†^ Excluded from multivariable comparison.

**Table 3.** Self-reported work and school attendance and absenteeism due to menstruation in Niamey, **Niger** according to socio-demographic characteristics and menstrual materials used

	Work (past month)			School (past year)		
	Worked (*n *= 978)	Missed (*n *= 212)	OR (95% CI)*	School (*n *= 375)	Missed (*n *= 213)	OR (95% CI)**
**Age**						
15–19	14.05	13.15	1.00 (0.70–1.42) (continuous)	58.10	10.08	1.17 (0.94–1.46) (continuous)
20–24	14.64	10.96		51.53	24.58	
25–29	33.11	6.56				
30–34	25.66	11.53				
35–39	25.32	15.92				
40–49	31.32	9.46				
**Marital status**						
In union	22.41	8.79	1.00	21.92	16.95	1.00
Not in union	22.07	13.02	1.55 (0.14–17.75)	63.18	14.59	0.84 (0.00–>100)
**Education**						
Never	15.83	0.0	–			
Primary	22.46	10.59	1.00	9.82	0.0	–
Secondary	19.24	11.50	1.10 (0.04–34.36)	62.57	15.47	1.00
Higher	46.51	17.60	1.80 (1.39–2.34)	80.98	13.83 (*n *= 4)	0.88 (<.00–>1000)
**Wealth tertile**						
Lowest tertile	19.20	15.18	1.00	41.61	10.98	1.00
Middle tertile	17.23	15.18	1.00 (0.26–3.84)	52.68	13.17	1.23 (0.03–41.18)
Highest tertile	28.73	6.48	0.39 (0.02–8.09)	67.37	17.14	1.68 (<.01–>100)
**Menstrual materials (multiple-response variable)**						
Pads	25.51	10.03	0.86 (0.49–1.50)	59.96	20.98	2.73 (<.01>100)
Cloth	19.59	5.57	0.42 (0.07–2.52)	51.20	2.97	0.15 (0.01–99.7)
Cotton wool	24.06	19.24	2.57 (<.01–>100)	55.81	9.48	0.55 (<.01>100)
Other (e.g. toilet paper, underwear)	21.30	12.62	1.24 (<.01–>100)	49.50	14.14	0.93 (0.12–7.05)
**Total**	**22.25**	**10.77**		**55.77**	**14.74**	

*Of respondents who reported working in the past month.

**Of respondents aged 15–24 who reported attending school in the past 12 months.

**Table 4.** Self-reported work and school attendance and absenteeism due to menstruation in **Nigeria** according to socio-demographic characteristics and menstrual materials used

	Work (past month)				School (past year)			
	Worked (*n *= 8360)	Missed (*n *= 3638)	OR (95%CI)*	aOR (95%CI)*	School (*n *= 2956)	Missed (*n *= 1574)	OR (95%CI)**	aOR (95%CI)**
**Age**								
15–19	28.34	29.16	1.00	1.00	68.12	21.89	1.00	1.00
20–24	46.18	22.16	0.69 (0.49–0.98)	0.84 (0.58–1.21)	30.03	28.24	1.04 (1.04–1.89)	1.28 (0.85–1.92)
25–29	52.24	19.10	0.57 (0.40–0.82)	0.80 (0.54–1.19)				
30–34	57.13	12.99	0.36 (0.25–0.53)	0.57 (0.37–0.89)				
35–39	58.78	10.70	0.29 (0.19–0.44)	0.49 (0.29–0.82)				
40–49	58.63	11.86	0.33 (0.22–-0.48)	0.53 (0.33–0.86)				
**Marital status**								
Married/Cohabitating	50.39	12.91	1.00	1.00	13.76	19.51	1.00	1.00
Divorced or widowed	57.52	14.74	1.17 (0.74–1.83)	1.12 (0.71–1.81)	32.16	2.44	0.10 (0.01–1.14)	0.11 (0.01–1.21)
Never married	45.17	23.80	2.11 (1.68–2.64)	1.73 (1.29–2.31)	61.16	23.67	1.28 (0.52–3.12)	1.20 (0.45–3.22)
**Education**								
Never	27.62	23.93	1.00	1.00	–	–	–	
Primary	39.23	19.07	0.75 (0.47–1.20)	0.80 (0.50–1.29)	23.24	15.14	1.00	1.00
Secondary	48.67	17.52	0.67 (0.42–1.08)	0.64 (0.40–1.04)	53.56	22.48	1.63 (0.80–3.31)	1.16 (0.56–2.38)
Higher	67.83	13.48	0.49 (0.30–0.82)	0.63 (0.36–1.09)	70.06	29.14	2.31 (1.07–4.97)	1.30 (0.55–3.05)
**Wealth tertile**								
Lowest tertile	31.84	23.76	1.00	1.00	44.16	15.48	1.00	1.00
Middle tertile	48.56	19.60	0.78 (0.50–1.22)	0.98 (0.65–1.49)	49.91	24.78	1.79 (1.08–2.99)	1.39 (0.74–2.61)
Highest tertile	59.43	12.85	0.47 (0.32–0.70)	0.76 (0.47–1.21)	59.96	25.71	1.89 (1.15–3.10)	1.28 (0.63–2.63)
**Residence**								
Rural	39.46	21.13	1.00	1.00	50.40	18.32	1.00	1.00
Urban	54.59	15.04	0.66 (0.45–0.97)	0.93 (0.61–1.41)	53.80	26.19	1.58 (1.10–2.28)	1.33 (0.83–2.12)
**Menstrual materials** (multiple response option variable)								
Pads	54.34	15.80	0.74 (0.54–1.01)	1.27 (0.87–1.85)	54.03	25.31	1.73 (1.10–2.72)	1.47 (0.88–2.46)
Cloth	36.14	23.16	1.71 (1.27–2.29)	1.62 (1.15–2.29)	48.33	18.32	0.68 (0.43–1.09)	1.23 (0.71–2.15)
Toilet paper	61.48	9.49	0.49 (0.29–0.84)	^†^	59.99	15.63	0.60 (0.27–1.33)	^†^
Other (e.g. cotton wool, underwear)	52.59	18.13	1.09 (0.64–1.87)	^†^	45.02	20.14	0.83 (0.33–2.06)	^†^
**State**								
Anambra	63.94	14.18	1.83 (1.19–2.81)	^†^	66.88	29.10	1.43 (0.78–2.60)	^†^
Kaduna	30.56	21.02	2.95 (1.82–4.78)	^†^	48.49	34.17	1.81 (0.98–3.36)	^†^
Kano	21.36	23.97	3.49 (1.79–6.82)	^†^	52.08	17.93	0.76 (0.42–1.40)	^†^
Lagos	62.24	8.28	1.00	^†^	47.33	22.27	1.00	^†^
Nasarawa	34.19	18.28	2.48 (1.45–4.24)	^†^	52.13	21.03	0.93 (0.54–1.61)	^†^
Rivers	64.27	22.58	3.23 (1.73–6.03)	^†^	55.46	24.01	1.10 (0.62–1.96)	^†^
Taraba	34.07	30.35	4.83 (2.77–8.42)	^†^	43.33	15.56	0.64 (0.23–1.82)	^†^
**Total**	**48.73**	**16.95**			**52.53**	**23.38**		

*Of respondents who reported working in the past month.

**Of respondents aged 15–24 who reported attending school in the past 12 months.

^†^ Excluded from multivariable comparison.

The proportion of respondents 15–49 years of age who undertook work outside the household ranged from 22% in Niger to 49% in Nigeria. A total of 53–56% of survey respondents aged 15–24 years reported having attended school in the past 12 months. The proportion of women and adolescent girls working outside the household increased in all countries with age, education and wealth. Greater proportions of urban respondents reported undertaking work in Nigeria and Burkina Faso. As wealth increased, so did the proportion of respondents (aged 15–24 years) reporting they had attended school in the past year in Niger and Nigeria.

### Work and school absence

The total proportion of women and adolescent girls reporting school or work absenteeism due to menstruation varied across contexts. Work absence due to menstruation during the last menstrual period was 11% in Niamey, Niger, 17% in Nigeria, and 19% in Burkina Faso. School absenteeism in the past year due to menstruation was also lowest in Niamey, Niger (15%), followed by Burkina Faso (17%), and highest (23%) in Nigeria. Contextual variations in the rates of absenteeism due to menstruation were also evident in state-level data in Nigeria where school absenteeism ranged from 16% in Taraba to 34% in Kaduna.

In Burkina Faso (Table 2), work absences due to menstruation were lower among those in urban compared to rural areas (13% vs 22%), among those with higher education (12% compared to 20% among those with no schooling) and for those in the highest wealth tertile (14%) compared to the lowest (24%). These differences were not statistically significant in binary or adjusted logistic regressions. A converse pattern was evident for absences from school due to menstruation, with those in urban areas and higher wealth tertiles reporting higher levels of absence. Again, these differences were not statistically significant. Among working respondents, 20% of those using cloth as menstrual absorbent reported absence due to menstruation compared to 14% of pad users, while among those attending school 18% of pad users reported absence due to menstruation and 15% of cloth users. However, material use was not a significant predictor of absence due to menstruation from school or work.

In Niamey, Niger (see Table 3), the small sample of those who had attended work in the past month or school in the last year meant demographic groupings were small and comparisons should be interpreted with caution. There were no statistically significant differences across demographic groups.

In Nigeria (see Table 4), binary relationships showed lower levels of absence from work due to menstruation among those with higher levels of education and wealth, and among urban residents. However, in multivariable models, only the use of cloth and never being married were significantly associated with increased odds of reporting absence from work in the last month due to menstruation. Among those attending school, an opposing relationship was present in the binary comparisons with higher levels of wealth, urban residence and sanitary pad use associated with higher proportions of absenteeism due to menstruation. While the differences in proportions appear meaningful (for example 24% reporting absence in the lowest wealth tertile compared to 13% among the wealthiest tertile), relationships were not statistically significant in multivariable comparisons, suggesting significant variability across the sample and considerable shared variance among socio-demographics.

## Discussion

This study provides the first representative estimates of school and work absenteeism due to menstruation in Burkina Faso, Nigeria and Niamey, Niger. Findings support mounting evidence for the impact of menstruation on girls’ education, and hypotheses that difficulties with menstruation influence females’ ability to undertake work outside the home.^7,10,11,34–36^

Among respondents who had worked outside the home in the past month in Burkina Faso, Niger and Nigeria, 19%, 11% and 17%, respectively, reported missing work due to menstruation. In Burkina Faso 17%, in Niamey, Niger 15% and in Nigeria 23% of respondents aged 15–24 years who had attended school in the past year reported absence due to menstruation. These figures are similar to the proportions of respondents who reported missing school, work, or social activities due to their last period in Sierra Leone, Ghana, and the Gambia MICS reports (approximately one in five), despite the different recall periods and combination of activities included in these estimates.^21,22,37^

The modest differences in absenteeism across the three West African countries in this study may be due to a range of contextual differences. Notably, data collection for Niger was only undertaken in an urban area, the capital Niamey. This may have contributed to the lower levels of work absenteeism compared to other countries, consistent with observed demographic trends. Proportions of absenteeism reported for each Nigerian state suggest a high level of regional variation. States with higher work absenteeism reported lower rates of school absence due to menstruation, consistent with the conflicting demographic trends observed within the country.

There were few statistically significant relationships between socio-demographics and self-reported absenteeism due to menstruation across countries. Self-reported absences due to menstruation typically varied by 5–15% of respondents across demographic groups. In general, women and adolescent girls with higher levels of wealth, education and urban residence reported less menstrual-related absences from work whereas students with these same characteristics reported more absences from school. These conflicting trends are challenging to interpret. We may hypothesise that those with higher levels of education and wealth may undertake employment which is less physically demanding. They may also have greater access to resources for pain relief, preferred menstrual materials such as commercially produced sanitary pads, and better access to soap and water. Any of these individual and workplace characteristics may help wealthier workers to remain at work while menstruating. We would also expect these same considerations to support school attendance during menstruation, but this trend was not observed across our three samples. One possible explanation is that workplaces may have greater variation in the demands of work activities, whereas schools are more uniform in their expectations. Students from greater socioeconomic advantage may have more freedom to remain at home if they are experiencing pain or discomfort during menstruation, while the relative cost of absence is greater for those who are more disadvantaged.

A possible explanation for the slightly higher absenteeism among more advantaged students is that the PMA2020 surveys only include respondents 15 years of age or older. By this age, more disadvantaged students may already have left school, and only those with greater support for education may remain. PMA2020 analytic samples only included family members who slept in the household the previous night, which excludes students attending boarding schools. Participants attending tertiary education were included in reported school absenteeism and may have more flexibility in their schedules to work around menstrual difficulties, although these represented a very small proportion of the respondents. We note that work and school absenteeism were assessed for different recall periods. When asking about work absenteeism, workers reported on absence in the past month. In contrast, students were asked about absenteeism from school during the last year. This time period was selected as students may not have attended school during the past month due to school holidays. However, there may be greater bias in recall over this longer period and this means that these proportions are not directly comparable.

The proportions of school and work absenteeism presented are limited by the sample eligible to answer these questions. Respondents were required to have had a menstrual period in the past three months to be asked questions related to menstruation. Past analyses have demonstrated that this sub-sample is likely to be urban and have greater levels of wealth and education than the full female questionnaire sample.^30^ Of this already-restricted sample, only half of the women and adolescent girls surveyed reported attending any school or work in the relevant time-periods and were thus asked about absences. This sample also had greater levels of resources than those not participating in school or work during the defined period. Thus, as an indicator for the impact of unmet menstrual needs, the proportions of absences reported in these nationally representative surveys do not represent the experiences of all women and girls. Future surveys using similar questions should note the significant loss of sample size due to eligibility criteria for their sample size calculations and consider the inclusion of questions that may apply to all women and girls such as impacts on social participation or daily activities. This approach was taken in recent MICS data collections where school and work absences were combined with missing other activities.^21–23^ However, while this approach prevents loss of respondents, it combines a wide range of activities into a single measure, resulting in a loss of specificity. The consequences of missed work in the home or social activities may be different from missing paid employment, and from missing school days. Consultation with governments and stakeholders may highlight further considerations for the usefulness of combined or separated outcomes.

The minimal statistically significant differences in absenteeism according to socio-demographic characteristics may also suggest that other, unmeasured characteristics are important drivers of absenteeism. Unmeasured characteristics such as internalised stigma around menstruation, social support and community expectations of behaviour may influence women’s and girls’ ability to attend to school and work during menstruation and may not be bounded by socio-demographic groupings. Importantly, unmeasured characteristics may also influence respondents’ propensity to recall or attribute absences to menstruation, biasing responses. Furthermore, menstrual pain or other symptoms associated with menstruation may be less likely to differ across socio-demographic groups. Across high-, middle-, and low-income countries, menstrual pain is frequently identified as a reason for school absenteeism. A study of 1051 high school students in Australia found that 26% of girls reported missing school due to menstruation, with 2% reporting missing school every period. Absences ranged from 1 to 4 days and increased with the level of pain reported.^38^ A 2016 study of 1300 high school girls in Turkey found that 32% reported missing school due to menstrual pain or heavy bleeding.^39^ In Mexico, 24% of girls experiencing dysmenorrhea reported missing school.^40^ The ENDOcost study which included women from 16 hospitals across 10 countries with diagnosed endometriosis, a condition affecting approximately 2–10% of the female population, found that these women lost an average 10.8 hours of work weekly.^41^ These figures suggest that even in settings where women and girls have greater access to safety-managed sanitation facilities,^42,43^ and many have resources to purchase commercial menstrual products, there remain high proportions of the population that miss school or work due to their periods. Advocates for greater gender sensitivity in the workplace have argued for increased flexibility in workplaces to support workers’ management of menstrual pain. This has provoked debate regarding the costs and benefits of dedicated menstrual leave policies. This perspective should inform responses to the absentee rates reported here and highlight limitations of the use of absenteeism as a primary indicator for menstrual health.

Thus, whilst the high proportions of school and work absenteeism reported here should provoke action from practitioners and policy makers, PMA2020 and similar national data alone are insufficient for informing policy decision making without supplementary questions or research in each context. These data do not indicate the reasons for menstrual absenteeism. Past research has indicated that a range of factors contribute to absences due to menstruation, including menstrual pain. Particularly in the Global South, difficulties managing menstrual bleeding have received greater attention than the burden of menstrual pain or disorders. This is despite past research highlighting the contribution of pain to absence from school during menstruation.^11,44,45^ The data as captured in the PMA2020 survey questions are unable to provide any indication of the relative contributions of these factors which would assist in informing policy or practice responses. Different intervention approaches may be needed if menstrual pain and disorders, rather than difficulties managing normal menstrual bleeding, account for a large proportion of absences.

## Conclusions

Our findings from nationally and city representative surveys support the assertion that menstruation is a source of absence from work and school in West Africa. The high rates of school and work absenteeism due to menstruation reported are cause for alarm and should direct greater attention and resources to this previously neglected part of life for women of reproductive age. The questions used in PMA2020 survey rounds in 2018 provide transparent, data-founded estimates of women’s and girls’ self-reported absenteeism. However, there are significant limitations to the use of these questions to track unmet menstrual health needs across countries. More work is needed to identify indicators best placed to inform decision making and we encourage continued critical reflection and revision of decisions taken.

## Ethical approvals

As a secondary data analysis of publicly available data, this study was exempt from review by the IRB of Johns Hopkins University Bloomberg School of Public Health. For the PMA2020 surveys, approval for human subjects research was granted by the following organisations in each country:

- **Burkina Faso:** Comite D’Ethique Pour La Recherche en Sante, Ministere de la Recherche Scientifique et de L’Innovation, Ministere de la Sante.
- **Niger:** Comité Consultatif National d’éthique.
- **Nigeria:** National Health Research Ethics Committee (NHREC), Department of Health Planning, Research and Statistics, Federal Ministry of Health.

## Data Availability

Data is publicly available for request on the PMA2020 website: https://www.pma2020.org/.

## Abbreviations

PMA2020: Performance Monitoring and Accountability 2020
EA: Enumeration Area
OR: Odds Ratio
aOR: adjusted Odds Ratio
CI: Confidence Interval
